# Identifying New Candidate Genes and Chemicals Related to Prostate Cancer Using a Hybrid Network and Shortest Path Approach

**DOI:** 10.1155/2015/462363

**Published:** 2015-10-04

**Authors:** Fei Yuan, You Zhou, Meng Wang, Jing Yang, Kai Wu, Changhong Lu, Xiangyin Kong, Yu-Dong Cai

**Affiliations:** ^1^Institute of Health Sciences, Shanghai Institutes for Biological Sciences, Chinese Academy of Sciences and Shanghai Jiao Tong University School of Medicine, Shanghai 200031, China; ^2^Department of Mathematics, East China Normal University, Shanghai 200241, China; ^3^College of Life Science, Shanghai University, Shanghai 200444, China

## Abstract

Prostate cancer is a type of cancer that occurs in the male prostate, a gland in the male reproductive system. Because prostate cancer cells may spread to other parts of the body and can influence human reproduction, understanding the mechanisms underlying this disease is critical for designing effective treatments. The identification of as many genes and chemicals related to prostate cancer as possible will enhance our understanding of this disease. In this study, we proposed a computational method to identify new candidate genes and chemicals based on currently known genes and chemicals related to prostate cancer by applying a shortest path approach in a hybrid network. The hybrid network was constructed according to information concerning chemical-chemical interactions, chemical-protein interactions, and protein-protein interactions. Many of the obtained genes and chemicals are associated with prostate cancer.

## 1. Introduction

The prostate is a gland in the male reproductive system that surrounds the prostatic urethra and affects urinary function. Its secretion is a component of semen. Prostate cancer is a form of adenocarcinoma. Most prostate cancers grow slowly, while some grow relatively rapidly [1, 2]. In the early stage, some prostate cancer patients present no symptoms, while others display symptoms similar to benign prostatic hyperplasia. Advanced prostate cancer can spread to other parts of the body, including the bones and lymph nodes [3]. Prostate cancer can also affect sexual function, such as erection and ejaculation. It is the world's second most common cancer [1]. More than 80% of men will be diagnosed with prostate cancer by the age of 80 [4], but, due to its slow growth, most patients do not die from this disease.

Biopsy is necessary to confirm the diagnosis of prostate cancer. Ultrasound (US) and magnetic resonance imaging (MRI) can help determine whether the cancer has metastasized [2]. Prostate specific antigen (PSA) screening is widely used in the USA to diagnose prostate cancer at an earlier age and cancer stage [5]. Noninvasive detection methods are being developed, including detecting EN2 and PCA3 mRNA in the urine [6, 7]. BCL-2, Ki-67, and ERK5 may also be useful as markers [8–10]. Treatment options for prostate cancer include surgery, radiation therapy, hormone therapy, and chemotherapy [2].

Prostate cancer risk is associated with age, family disease history, and race. It is not monogenic; many genes are involved. For example, mutations in BRCA1 and BRCA2 have been implicated in prostate cancer, while they are also risk factors for ovarian cancer and breast cancer [11]. p53 mutations are more frequently observed after prostate cancer metastasis. Additionally, one copy of the tumor suppressor gene PTEN is lost in up to 70% of prostate cancer patients [12]. Genome-wide association studies have identified several SNPs that affect prostate cancer risk [13–15]. The transcription factor RUNX2 can prevent prostate cancer cell apoptosis [16], and inhibition of X-linked inhibitor of apoptosis (XIAP) is being studied as a strategy to enhance apoptosis and prevent cancer cell proliferation [17]. Sexually transmissible infections (STI), such as HPV-16, HPV-18, and HSV-2, are significantly linked with prostate cancer [18–20].

Several chemicals have also been studied in prostate cancer. Zinc can change prostate cell metabolism to produce citrate, an important component of semen. This process requires a large amount of energy and prostate cancer cells that are devoid of zinc reserve energy for growth [21]. The prostate glands require androgens to work properly. Hormone therapies, including castration treatment (reduction of androgen/testosterone/DHT), are commonly used, but they are only effective in a subset of patients. Androgen receptor inhibition is effective in mouse studies [22]. More treatments are being tested to improve the survival of castration-resistant prostate cancer patients.

As discussed above, prostate cancer is a very complicated disease, and we have yet to identify all risk factors. Additional genes and chemicals remain to be discovered. While it is time consuming and expensive to identify genes or chemicals related to prostate cancer using traditional approaches, the development of computer science can overcome these obstacles by building effective computational methods. Here, we proposed an alternative computational method to identify new candidate genes and chemicals related to prostate cancer. To simultaneously investigate genes and chemicals, a hybrid network was constructed using chemical-chemical interactions and chemical-protein interactions from STITCH (search tool for interactions of chemicals) [23] and protein-protein interactions from STRING (search tool for the retrieval of interacting genes/proteins) [24]. By applying a shortest path approach in the hybrid network, we extracted genes and chemicals related to prostate cancer. To validate our model, several of the identified genes and chemicals were investigated in related prostate cancer literature.

## 2. Materials and Methods

### 2.1. Genes Related to Prostate Cancer

We collected genes related to prostate cancer using the following approaches: (I) 143 reviewed genes were chosen from UniProt (http://www.uniprot.org/, UniProt Release 2014_4) [25] using the search terms, “human,” “prostatic cancer,” and “reviewed”; (II) 86 genes were chosen from the TSGene Database (Tumor Suppressor Gene Database, http://bioinfo.mc.vanderbilt.edu/TSGene/cancer_type.cgi [26]) after the Entrez IDs were converted into their official symbols; and (III) 96 genes were retrieved from the NCI (National Cancer Institute, https://gforge.nci.nih.gov, released 2009.6) database [27]. After integrating the aforementioned 325 genes, we obtained 309 genes related to prostate cancer (Supplementary Material I; see Supplementary Material available online at http://dx.doi.org/10.1155/2015/462363).

### 2.2. Chemicals Related to Prostate Cancer

Chemicals related to prostate cancer were collected from the CTD (Comparative Toxicogenomics Database) (http://ctdbase.org/detail.go?type=disease&acc=MESH:D011471&view=chem, July 2014) [28]. These chemicals were manually assessed in the literature. Here, 177 chemicals with direct evidence of association with prostate cancer, such as “marker,” “mechanism,” or “therapeutic,” were considered. Among these 177 chemicals, 106 were present in the hybrid network described below (see Section 2.3). Thus, we employed these 106 chemicals in this study (Supplementary Material I).

### 2.3. Hybrid Network

The hybrid network was constructed according to information based on chemical-chemical interactions, chemical-protein interactions, and protein-protein interactions. In brief, the chemical-chemical interactions and chemical-protein interactions were retrieved from STITCH (version 4.0, http://stitch.embl.de/) [23], and the protein-protein interactions were downloaded from STRING (version 9.1, http://www.string-db.org/) [24]. The obtained interactions include both known and predicted interactions. Thus, they can widely measure the associations between chemicals and proteins, and they have been widely used to investigate many chemical-related and protein-related problems [29–40]. In addition, to measure the strength of these interactions, each interaction was assigned a score in STITCH and STRING. The score of the chemical-chemical interaction between chemicals *c*_1_ and *c*_2_ was denoted by *S*_*cc*_(*c*_1_, *c*_2_), the score of the chemical-protein interaction between chemical *c* and protein *p* by *S*_*cp*_(*c*, *p*), and the score of the protein-protein interaction between proteins *p*_1_ and *p*_2_ by *S*_*pp*_(*p*_1_, *p*_2_). Due to the large number of chemicals, we only considered chemicals with KEGG (Kyoto Encyclopedia of Genes and Genomes) records [41] to reduce search space (i.e., chemicals occurring in the retrieved chemical-protein interactions and chemical-chemical interactions must be in KEGG).

The hybrid network used proteins and chemicals from the three types of interactions as nodes. Each edge represented one of the three types of interactions, and they were assigned a weight to indicate the strength of the interaction using the following equations:

(1) $$\begin{array}{c} w\left(e\right)=\left\{\begin{array}{cc} 1000-S_{pp}\left(p_{1},p_{2}\right) & \text{If}\;n_{1}\;\text{and}\;n_{2}\;\text{represented}\\ & \text{proteins}\;p_{1}\;\text{and}\;p_{2}\\ 1000-S_{cp}\left(c,p\right) & \text{If}\;n_{1}\;\text{and}\;n_{2}\;\text{represented}\\ & \text{chemical}\;c\;\text{and}\;\text{protein}\;p\\ 1000-S_{cc}\left(c_{1},c_{2}\right) & \text{If}\;n_{1}\;\text{and}\;n_{2}\;\text{represented}\\ & \text{chemicals}\;c_{1}\;\text{and}\;c_{2}. \end{array}\right. \end{array}$$

Finally, we obtained a hybrid network consisting of 35,842 nodes, where 15,072 nodes represented chemicals and 20,770 nodes represented proteins. The size of the network, that is, the number of edges in the network, was 3,046,625, where 398,701 edges represented chemical-chemical interactions, 222,610 edges represented chemical-protein interactions, and 2,425,314 edges represented protein-protein interactions.

### 2.4. A Shortest Path Approach Used to Identify New Candidate Genes and Chemicals

Chemicals or proteins that comprise an interaction always have similar functions [31, 36, 42]. One chemical/protein and one chemical/protein that interact with a high score (low weight of the corresponding edge in the hybrid network) are more likely to share similar functions than those with a low score. Therefore, we can infer that chemicals/proteins occurring in a shortest path connecting the chemicals/proteins, *n*_1_ and *n*_2_, are likely to share functions with *n*_1_ and *n*_2_. Thus, we searched all the shortest paths connecting any pair of chemicals and proteins related to prostate cancer, and the corresponding chemicals and proteins occurring in these paths were considered candidate chemicals and genes. Simultaneously, the number of paths containing a certain candidate chemical or gene was termed “betweenness.”

Some of the candidate chemicals and genes may be false positives, and some chemicals or proteins may have universal associations with other chemicals or proteins, so they are observed in the shortest paths connecting any pair of randomly selected chemicals or proteins. To control for these false positives, we randomly produced 1,000 chemical and protein sets, and each set had the same numbers of chemicals and proteins as the set consisting of chemicals and genes related to prostate cancer. For each set, we searched for the shortest paths connecting any pair of chemicals or proteins and counted the betweenness of the candidate chemicals and proteins based on these paths. Then, we counted the number of randomly produced sets in which the betweenness was larger than the set consisting of chemicals and genes related to prostate cancer for each candidate chemical or gene; the *P* value was defined as the aforementioned number divided by 1,000. Thus, a low *P* value for a certain candidate chemical or gene indicates strong linkage with prostate cancer.

## 3. Results and Discussion

### 3.1. Candidate Genes and Chemicals

As mentioned in Sections 2.1 and 2.2, we employed 309 genes and 106 chemicals related to prostate cancer. We searched all shortest paths connecting any of these genes. Based on the obtained paths, we extracted 595 candidate genes and 102 candidate chemicals and calculated their betweenness (Supplementary Material II). According to the method in Section 2.4, the *P* values of these candidate genes and chemicals were computed to control for false positives, which are also listed in Supplementary Material II. Then, we set the *P* value threshold as 0.05 to select for significant candidate genes and chemicals (i.e., candidate genes and chemicals with *P* values less than 0.05 were selected). Ultimately, 187 genes and 11 chemicals were selected (Supplementary Material III).

### 3.2. Analysis of Enriched KEGG Pathways of Significant Candidate Genes

As mentioned in Section 3.1, we obtained 187 significant candidate genes that were potentially related to prostate cancer pathogenesis. To analyze the relationship between these genes and prostate cancer, we employed a functional annotation tool, DAVID (Database for Annotation, Visualization and Integrated Discovery) [43], to understand their biological significance. The results of DAVID included the enrichment of the 187 significant candidate genes in KEGG pathways and GO terms (Supplementary Material IV and V, resp.).

**Figure 1:**
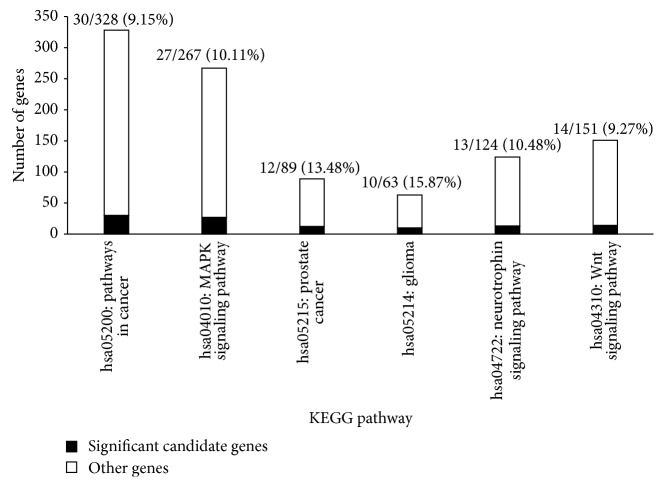
Top six pathways highly associated with prostate cancer analyzed by DAVID. The black part represents the number of significant candidate genes sharing the pathway; the white part represents the number of other genes sharing the pathway.

In total, the 187 significant candidate genes shared 40 KEGG pathways. After sorting the 40 KEGG pathways according to their FDR (false discovery rate) adjusted *P* value (last column in Supplementary Material IV), we found that the top six pathways were highly associated with prostate cancer.  Figure 1 shows these pathways, the number of genes among the 187 significant candidate genes that shared each pathway and the proportion of these genes among all genes sharing the pathway.  Table 1 lists the FDR of these pathways.

The most enriched pathway was hsa05200: pathways in cancer, with 30 significant candidate genes sharing this pathway (see Figure 1) and an FDR of 2.08*E* − 06 (see Table 1, row 2). The fourth most enriched pathway was hsa05214: glioma, with 10 significant candidate genes sharing this pathway (see Figure 1) and an FDR of 3.03*E* − 02 (see Table 1, row 5). These results indicate that prostate cancer and other types of cancer share a common mechanism.

The second most enriched pathway was hsa04010: MAPK signaling pathway, with 27 significant candidate genes (see Figure 1) and an FDR of 2.15*E* − 06 (see Table 1, row 3). Mitogen-activated protein kinase (MAPK) pathways are evolutionarily conserved and link extracellular signals to fundamental cellular processes. Mutations in these pathways can affect Ras and B-Raf and play a critical role in cancer development [44].

The third most enriched pathway was hsa05215: prostate cancer, with 12 significant candidate genes (see Figure 1) and an FDR of 1.56*E* − 02 (see Table 1, row 4). This result shows that some of the candidate genes have already been grouped into the pathway which was drawn based on the previous knowledge of molecular interaction and reaction networks in prostate cancer.

The fifth most enriched pathway was hsa04722: neurotrophin signaling pathway, with 13 significant candidate genes (see Figure 1) and an FDR of 7.59*E* − 02 (see Table 1, row 6). Neurotrophins play a role in the survival of malignant prostate cells [45]. Neurotrophins include nerve growth factor (NGF), brain-derived neurotrophic factor (BDNF), neurotrophin 3 (NT-3), and neurotrophin 4/5 (NT4/5), and they bind with trk receptors. The survival of malignant prostate cells requires ectopic expression of trk B and trk C and continued expression of trk A. Trk inhibition has been suggested to be a drug therapeutic target [46].

The sixth most enriched pathway was hsa04310: Wnt signaling pathway, with 14 significant candidate genes (see Figure 1) and an FDR of 1.26*E* − 01 (see Table 1, row 7). The Wnt signaling pathway is involved in carcinogenesis and embryonic development. It acts as a common element in the regulation of stem cell renewal and the maintenance of many cellular systems. Disruption of this pathway is associated with cancer [47]. Mutations in components of this pathway, including APC, Axin, Axin2/conduction, and *β*-catenin, are found in a variety of cancers [48]. The Wnt signaling pathway plays a critical role in prostate cancer, as its key component, *β*-catenin, works as an androgen receptor (AR) cofactor. *β*-Catenin can significantly enhance androgen-stimulated transcriptional activation by the AR [49]. Abnormal expression of Wnt ligands and receptors may also contribute to the pathogenesis of prostate cancer [50].

### 3.3. Analysis of Enriched GO Terms of Significant Candidate Genes

**Figure 2:**
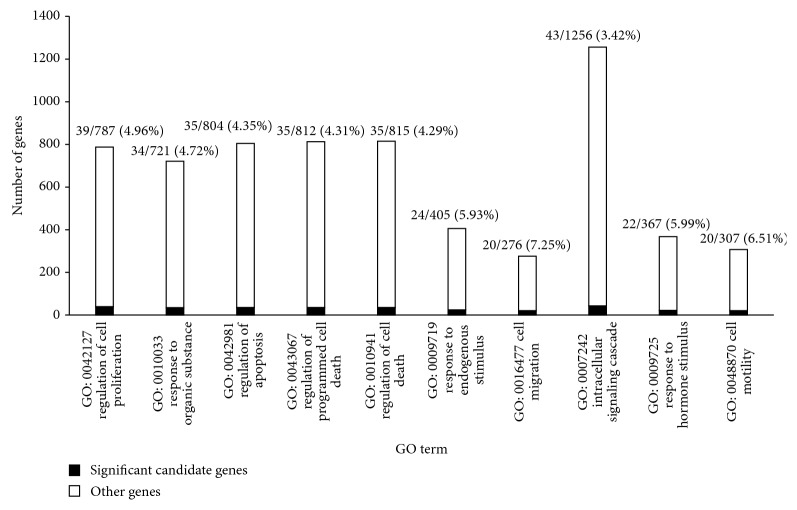
Top ten GO terms highly related to prostate cancer analyzed by DAVID. The black part represents the number of significant candidate genes sharing the GO term; the white part represents the number of other genes sharing the GO term.

In total, the 187 significant candidate genes enriched 576 GO terms (Supplementary Material V), and we investigated the top ten GO terms sorted by FDR.  Figure 2 shows these GO terms, the number of genes among the 187 significant candidate genes that shared each GO term and the proportion of these genes among all genes sharing the GO term.  Table 2 lists the FDR of these GO terms.

All of these ten GO terms were biological process (BP) GO terms, and four were associated with the regulation of cell proliferation and death: GO:0042127, regulation of cell proliferation (39 significant candidate genes sharing this GO term, refer to Figure 2) (“FDR” = 2.11*E* − 09, refer to Table 2); GO:0042981, regulation of apoptosis (35 significant candidate genes sharing this GO term, refer to Figure 2) (“FDR” = 1.38*E* − 06, refer to Table 2); GO:0043067, regulation of programmed cell death (35 significant candidate genes sharing this GO term, refer to Figure 2) (“FDR” = 1.79*E* − 06, refer to Table 2); and GO:0010941, regulation of cell death (35 significant candidate genes sharing this GO term, refer to Figure 2) (“FDR” = 1.97*E* − 06, refer to Table 2). Cell proliferation and apoptosis are both important biological processes that may lead to cancer if altered by gene mutation and other risk factors. An increasing number of studies have demonstrated that important genes and miRNAs that participate in these processes could be therapeutic targets. For instance, miR-145 functions as a tumor suppressor. By targeting FSCN1, miR-145 suppresses cell proliferation in prostate cancer, and it represents an important therapeutic target [51].

Three GO terms were associated with cell responses to stimulus: GO:0010033, response to organic substance (34 significant candidate genes sharing this GO term, refer to Figure 2) (“FDR” = 3.38*E* − 07, refer to Table 2); GO:0009719, response to endogenous stimulus 24 significant candidate genes sharing this GO term, refer to Figure 2) (“FDR” = 4.75*E* − 06, refer to Table 2); and GO:0009725, response to hormone stimulus (22 significant candidate genes sharing this GO term, refer to Figure 2) (“FDR” = 2.16*E* − 05, refer to Table 2). Sex hormones play an important role in the growth and development of the prostate [52]. Testosterone is implicated in the pathogenesis of prostate cancer [53]. Hormone therapy is currently used in the clinical treatment of prostate cancer, but it is only effective in a subset of patients. A recent study found no association between prediagnostic circulating sex hormones and lethal prostate cancer or total mortality [54]. This topic remains debatable, and further prospective studies are needed. Small chemicals that can stimulate prostate cells also warrant further attention.

Two GO terms were associated with cell motility: GO:0016477, cell migration (20 significant candidate genes sharing this GO term, refer to Figure 2) (“FDR” = 5.61*E* − 06, refer to Table 2), and GO:0048870, cell motility (20 significant candidate genes sharing this GO term, refer to Figure 2) (“FDR” = 3.20*E* − 05, refer to Table 2). Metastatic prostate cancer often spreads to bone, but the lung and liver are also common sites. More symptoms may occur depending on the site of cancer spread.

The last term was GO:0007242: intracellular signaling cascade (43 significant candidate genes sharing this GO term, refer to Figure 2) (“FDR” = 1.26*E* − 05, refer to Table 2). A recent report demonstrated that activation of Stat3 signaling was essential for prostate cancer progression, and inhibition of this pathway may be a therapeutic strategy [55]. Downregulation of Notch-1 and Jagged-1 could inhibit prostate cancer cell growth, migration and invasion, and induce apoptosis via inactivation of the Akt, mTOR, and NF-*κ*B signaling pathways [56].

### 3.4. Analysis of Significant Candidate Genes

In our study, 187 significant candidate genes were obtained (Supplementary Material III), where 42 genes were with *P* value 0. Among these 42 genes, 21 genes were found to be reported as prostate cancer related genes in some previous studies, which implies our method is quite effective. Please see Table 3 for the detailed information of these 21 genes. For the rest 21 significant candidate genes with *P* value 0, four of them (listed in rows 2–5 of Table 4) were deemed to be related to prostate cancer based on their current validated functions. They were discussed as below.

*PLCG1*. PLCG1 (phospholipase C, gamma 1) encodes the enzyme required to catalyze the formation of inositol IP3 (1,4,5-trisphosphate) and DAG (diacylglycerol) from phosphatidylinositol 4,5-bisphosphate. In this process, IP3 uses Ca^2+^ as a cofactor for nuclear translocation and the subsequent activation of downstream targets [57]. In our study, PLCG1 was highly related to prostate cancer, as demonstrated by its high betweenness (2,110; see row 2 of Table 4) and low *P* value (0; see row 2 of Table 4). Frequent mutations occur in the catalytic domain of PLCG1, which induce the activation of downstream signaling pathway and PLCG1 was sensitive to specific inhibition of CaN in CTCL (cutaneous T-cell lymphoma) [58]. Many receptors, such as EGF (epidermal growth factor) and PDGF (platelet-derived growth factor), are affected by PLCG1 [59, 60]. In addition, PLCG1 plays a key role in chemotaxis triggered by growth factor receptors, and it is involved in integrin-dependent cell motility in diverse types of cancer [61]. Research regarding the function of PLCG1 in prostate cancer is rare; we remind that PLCG1 is a diagnostic marker and a drug target in prostate cancer.

*BIRC2*. BIRC2 (baculoviral IAP repeat containing 2), also known as API1 or cIAP1 (cellular inhibitors of apoptosis), belongs to a protein family that binds TRAF1/2 (tumor necrosis factor receptor-associated factors) to inhibit apoptosis. In our study, BIRC2 was closely associated with human prostate cancer, and its betweenness and *P* value were 1,583 and 0, respectively (see row 3 of Table 4). ARC (caspase recruitment) regulates BIRC2, and BIRC2 expression is inverse to ARC in AML (acute myeloid leukemia) [62, 63]. In addition, in metastatic human colon and breast cancer cells, BIRC2 is the molecular target of ceramide, and the Smac mimetic, BV6, targets BIRC2 to induce apoptosis via the TNF*α* signaling pathway [64, 65]. However, the detailed mechanism of BIRC2 action remains unknown. We speculate that BIRC2 is a key apoptosis-associated factor in prostate cancer that warrants further experimentation.

*AMELY*. In prostate cancer, many driver genes are gender-related. In our study, a gender-related locus gene, AMELY (amelogenin Y-linked) (betweenness: 363, *P* value: 0; see row 4 of Table 4), was related to prostate cancer. AMELY, which belongs to the amelogenin family of extracellular matrix proteins, is a single copy gene locus on the Y chromosome (Yp11.2) [66, 67]. AMELY and its homolog, AMELX, are often used for gender identification [68]. Deletions of AMELY occur frequently in certain ethnic populations [69–71]. Research regarding AMELY function is rare, especially in human prostate cancer, but we believe that it may be a potential gender-related gene and a biomarker in human prostate cancer. In the future, more experiments and clinical samples are still needed to validate the importance of this gene in prostate cancer.

*ELL*. ELL, the eleven-nineteen lysine-rich leukemia gene, encodes an RNA polymerase II transcription elongation factor that suppresses transient pausing by RNA polymerase II and functions in the process of transcription [72–74]. ELL was significantly associated with prostate cancer, as demonstrated by its high betweenness (363, see row 5 of Table 4) and low *P* value (0, see row 5 of Table 4). ELL was initially identified as a partner gene fused to MLL in the t(11;19) (q23; p13.1) translocation in AML (acute myeloid leukemia) [75]. U19/Eaf2 is an androgen-response gene that forms nuclear speckles by binding to ELL* in vivo*. U19/Eaf2 is downregulated in human prostate cancer, and its overexpression induces prostate cancer cell apoptosis [76]. Direct evidence regarding the function of ELL in human prostate cancer is rare, but our data and previous studies suggest that ELL is an inducer of apoptosis and a putative target in human prostate cancer.

Besides, significant candidate genes that were not discussed here still may be related to prostate cancer. We listed them in Supplementary Material III and hope that they will be the useful information for further study on prostate cancer.

### 3.5. Analysis of Significant Candidate Chemicals

We also obtained 11 significant candidate chemicals involved in prostate cancer (Supplementary Material III). This section discusses the relationships between several candidate chemicals and prostate cancer. Information pertaining to the discussed chemicals is listed in rows 6–10 of Table 4.

*Caffeine*. The betweenness and *P* value of caffeine (PubChem ID: CID000002519) were 371 and 0.028, respectively (row 6 of Table 4). Caffeine is a bitter, white crystalline xanthine alkaloid that can be extracted from coffee, tea, and other sources. A complex relationship has been reported between caffeine and cancer. For example, Sarkaria et al. suggested that caffeine could cause checkpoint defects, and, as a result, it might be useful for cancer therapy [77]. This statement could be regarded as an evidence to support our result. However, Wilson et al. observed a strong inverse association between coffee consumption and the risk of lethal prostate cancer, but this association appeared to be related to noncaffeine components of coffee [78]. Michels et al. did not find a strong association between caffeine and colon or rectal cancer [79]. Thus, further studies are needed to determine whether caffeine is associated with prostate cancer.

*Trifluoperazine*. The betweenness and *P* value of trifluoperazine (PubChem ID: CID000005566) were 363 and 0.001, respectively (row 7 of Table 4). Trifluoperazine is a typical antipsychotic medicine of the phenothiazine chemical class. Calmodulin (CaM) is critical for the proliferation and viability of cells, including cancer cells. Trifluoperazine inhibits CaM [80]. The antitumor properties of trifluoperazine have been reported in murine T-cell lymphomas, metastatic breast cancer, and prostatic cancer [81–84]. These reports support the robustness of our analysis.

*Mibefradil*. The betweenness and *P*-value of mibefradil (PubChem ID: CID000060662) were 363 and 0.013, respectively (row 8 of Table 4). Mibefradil is a blocker of the L/T-type calcium channel [85], which plays an essential role in regulating cell growth and proliferation [86]. Dysregulation of this channel may lead to tumor progression [87]. Blocking the T-type Ca^2+^ channel with mibefradil inhibits tumor cell proliferation and migration in multiple types of tumors, including human astrocytoma, neuroblastoma, glioblastoma, and breast cancer cells [85, 87–89]. Our results suggest that mibefradil represents a new candidate chemical for prostate cancer.

*Icilin*. The betweenness and *P* value of icilin (PubChem ID: CID000161930) were 363 and 0, respectively (row 9 of Table 4). Icilin is an artificial superagonist of the transient receptor potential M8 (TRPM8) ion channel. Cold and cooling agents activate TRPM8, inducing a cooling sensation. TRPM8 is a tumor marker for diagnosis and a target for cancer therapy. TRPM8 expression increases in the early stages of prostate cancer, and it is involved in prostate cell apoptosis [90]. Direct activation of TRPM8 by icilin inhibits prostate cancer by reducing cancer cell motility [91]. Taken together with previous studies, our results suggest that icilin is closely related to prostate cancer, and it may be a promising drug.

*Allicin*. The betweenness and *P* value of allicin (PubChem ID: CID000065036) were 2 and 0.024, respectively (row 10 of Table 4). Allicin is a garlic extract with antibacterial properties. The antitumor ability of allicin can be traced back to the early 1960s [92]. Currently, many studies have reported that garlic and its extracts can prevent cancer, such as skin cancer [93], hepatocarcinoma [94], and so forth [95, 96]. Garlic may work by enhancing repair DNA synthesis (RDS), depressing nitrosamine formation and reducing carcinogen bioactivation [97, 98]. The correlation between allicin and prostate cancer may provide novel insight for future research.

## 4. Conclusions

This work provided an alternative computational method to investigate prostate cancer. Several candidate genes and chemicals were extracted using this method, and analysis of the literature confirmed that they are related to prostate cancer. We hope that the results of this study will lead to the validation of these genes and chemicals.

## Supplementary Material

The Supplementary Material contains five files. In detail, Supplementary Material I lists genes and chemicals related to prostate cancer; Supplementary Material II lists candidate genes and chemicals, their betweenness and p-values; Supplementary Material III lists significant candidate genes and chemicals, their betweenness and p-values; Supplementary Material IV lists KEGG enrichment results of 187 significant candidate genes; Supplementary Material V lists GO enrichment results of 187 significant candidate genes.

## Figures and Tables

**Table 1:** The top six KEGG pathways shared by 187 significant candidate genes.

Pathway ID	Pathway name	Genes sharing the pathway	FDR
hsa05200	Pathways in cancer	FGF6, FGFR2, TRAF2, FGFR1, PDGFB, WNT3A, MITF, NFKB1, TGFB1, CTNNB1, GLI1, MAX, WNT1, CASP3, RARA, HHIP, AXIN1, PIK3R2, CREBBP, CDK6, BIRC2, RALGDS, CCND1, PLCG1, NTRK1, MAPK3, PDGFRB, PTCH1, IKBKB, and GSTP1	2.08*E* − 06

hsa04010	MAPK signaling pathway	FGFR2, FGF6, TRAF2, FGFR1, PDGFB, PPP3R1, NFKB1, TGFB1, ATF2, MAP3K7, MAX, TNFRSF1A, CASP3, MAP3K5, MAP3K3, MAP2K6, RASA1, FLNA, MAPK14, NTRK1, GADD45G, MAPK3, PDGFRB, HSPB1, IKBKB, CD14, and DUSP6	2.15*E* − 06

hsa05215	Prostate cancer	FGFR2, FGFR1, CCND1, PDGFB, MAPK3, CREBBP, PDGFRB, NFKB1, IKBKB, GSTP1, CTNNB1, and PIK3R2	1.56*E* − 02

hsa05214	Glioma	CCND1, PLCG1, PDGFB, CAMK2G, MAPK3, PDGFRB, CDK6, SHC1, CALM2, and PIK3R2	3.03*E* − 02

hsa04722	Neurotrophin signaling pathway	CAMK2G, NFKB1, MAGED1, MAP3K5, MAP3K3, PLCG1, NTRK1, MAPK14, MAPK3, SHC1, IKBKB, CALM2, and PIK3R2	7.59*E* − 02

hsa04310	Wnt signaling pathway	ROCK1, WNT3A, CAMK2G, CREBBP, CSNK2B, PPP3R1, CTNNB1, MAP3K7, WNT1, CCND1, CSNK1E, LRP6, LRP5, and AXIN1	1.26*E* − 01

**Table 2:** The top ten GO terms shared by 187 significant candidate genes.

GO term ID	GO term	Genes sharing the GO term	FDR
GO:0042127	Regulation of cell proliferation	FGFR2, FGFR1, CCL2, PDGFB, NDN, MITF, STRN, GNRHR, VIPR1, FOXO4, GHRHR, TGFB1, CTNNB1, GLI1, MAGED1, CTTNBP2, VDR, CASP3, MYOCD, SFTPD, SHC1, MUC2, PTGER2, GNRH1, CDK6, LIG4, DBH, NTN1, CDKN1C, PRKCQ, CCND1, HNF4A, HGS, TGFBR3, PDGFRB, PTCH1, SST, ADRA1D, and LRP5	2.11*E* − 09

GO:0010033	Response to organic substance	CGA, CCL2, PDGFB, LHCGR, NR3C1, FOXO4, TGFB1, GHRHR, CTNNB1, B2M, CTTNBP2, TNFRSF1A, CASP3, REN, RARA, SHC1, KCNMA1, GNRH1, CSNK2B, ESR1, DBH, BIRC2, PRKCQ, CCND1, HNF4A, MAPK14, ALDH2, HSD11B2, HSPB1, TGFBR3, PTCH1, IRF3, SST, and CD14	3.38*E* − 07

GO:0042981	Regulation of apoptosis	TRAF2, C9, CCL2, MITF, PPP3R1, NFKB1, RRM2B, NR3C1, TGFB1, MAGED1, MAP3K7, VDR, BAK1, MAP3K5, CASP3, NQO1, TERT, MAP2K6, RASA1, TERF1, KCNMA1, MUC2, GNRH1, ROCK1, ESR1, LIG4, DBH, BIRC2, TNFRSF10B, NTRK1, UBC, HSPB1, IKBKB, SST, and GSTP1	1.38*E* − 06

GO:0043067	Regulation of programmed cell death	TRAF2, C9, CCL2, MITF, PPP3R1, NFKB1, RRM2B, NR3C1, TGFB1, MAGED1, MAP3K7, VDR, BAK1, MAP3K5, CASP3, NQO1, TERT, MAP2K6, RASA1, TERF1, KCNMA1, MUC2, GNRH1, ROCK1, ESR1, LIG4, DBH, BIRC2, TNFRSF10B, NTRK1, UBC, HSPB1, IKBKB, SST, and GSTP1	1.79*E* − 06

GO:0010941	Regulation of cell death	TRAF2, C9, CCL2, MITF, PPP3R1, NFKB1, RRM2B, NR3C1, TGFB1, MAGED1, MAP3K7, VDR, BAK1, MAP3K5, CASP3, NQO1, TERT, MAP2K6, RASA1, TERF1, KCNMA1, MUC2, GNRH1, ROCK1, ESR1, LIG4, DBH, BIRC2, TNFRSF10B, NTRK1, UBC, HSPB1, IKBKB, SST, and GSTP1	1.97*E* − 06

GO:0009719	Response to endogenous stimulus	KCNMA1, CGA, CCL2, GNRH1, PDGFB, LHCGR, ESR1, FOXO4, DBH, BIRC2, TGFB1, GHRHR, CTNNB1, CTTNBP2, PRKCQ, CCND1, REN, ALDH2, TGFBR3, HSD11B2, RARA, SHC1, PTCH1, and SST	4.75*E* − 06

GO:0016477	Cell migration	ICAM1, CCL2, ROCK1, PDGFB, NDN, NUP85, CDH2, CX3CL1, DBH, NTN1, TGFB1, CTTNBP2, WNT1, CKLF, LRP6, SFTPD, TGFBR3, PDGFRB, SCNN1B, and LRP5	5.61*E* − 06

GO:0007242	Intracellular signaling cascade	TRAF2, FGFR1, CYP24A1, CCL2, LHCGR, NR3C1, VIPR1, FOXO4, GHRHR, CTNNB1, MAP3K7, VDR, MAP3K5, MAP3K3, REN, RARA, SHC1, RASA1, MAP2K6, CNKSR1, CCM2, ROCK1, ESR1, RALGDS, FLNA, PRKCQ, CCND1, NCOA1, TNFRSF10B, PLCG1, NEDD4, MAPK14, NTRK1, KRIT1, GADD45G, MAPK3, RAB5A, TGFBR3, IRF3, IKBKB, ADRA1D, GRB14, DUSP6	1.26*E* − 05

GO:0009725	Response to hormone stimulus	KCNMA1, CGA, CCL2, GNRH1, PDGFB, LHCGR, ESR1, FOXO4, GHRHR, TGFB1, CTNNB1, CTTNBP2, PRKCQ, CCND1, REN, ALDH2, TGFBR3, HSD11B2, RARA, SHC1, PTCH1, SST	2.16*E* − 05

GO:0048870	Cell motility	ICAM1, CCL2, ROCK1, PDGFB, NDN, NUP85, CDH2, CX3CL1, DBH, NTN1, TGFB1, CTTNBP2, WNT1, CKLF, LRP6, SFTPD, TGFBR3, PDGFRB, SCNN1B, LRP5	3.20*E* − 05

**Table 3:** 21 significant candidate genes with *P* value 0 which have been reported to be related to prostate cancer in previous studies.

Gene ID	Gene name	Betweenness	*P* value	Supporting references
ENSP00000320940	NCOA1	3886	0	[99]
ENSP00000262367	CREBBP	3569	0	[100]
ENSP00000340858	B2M	2564	0	[101]
ENSP00000287641	SST	1085	0	[102]
ENSP00000410294	FGFR2	1085	0	[103]
ENSP00000346294	S100A4	365	0	[104]
ENSP00000226413	GNRHR	363	0	[105]
ENSP00000263408	C9	363	0	[106]
ENSP00000264001	CKLF	363	0	[107]
ENSP00000293308	KRT8	363	0	[108]
ENSP00000294954	LHCGR	363	0	[109]
ENSP00000298772	TRIM13	363	0	[110]
ENSP00000330382	PDGFB	363	0	[111]
ENSP00000348775	ACOX3	363	0	[112]
ENSP00000361366	SFTPD	363	0	[113]
ENSP00000382166	CX3CR1	363	0	[114]
ENSP00000413720	CDKN1C	363	0	[115]
ENSP00000216862	CYP24A1	36	0	[116]
ENSP00000420168	GSTA2	20	0	[117]
ENSP00000276431	TNFRSF10B	11	0	[118]
ENSP00000263946	PKP1	1	0	[119]

**Table 4:** Information regarding significant candidate genes and chemicals related to prostate cancer.

Gene or chemical ID	Gene or chemical name	Betweenness	*P* value
ENSP00000244007	PLCG1	2,110	0
ENSP00000227758	BIRC2	1,583	0
ENSP00000215479	AMELY	363	0
ENSP00000262809	ELL	363	0
CID000002519	Caffeine	371	0.028
CID000005566	Trifluoperazine	363	0.001
CID000060662	Mibefradil	363	0.013
CID000161930	Icilin	363	0
CID000065036	Allicin	2	0.024
