# Supplementary material for: Identifying New Candidate Genes and Chemicals Related to Prostate Cancer Using a Hybrid Network and Shortest Path Approach
Source: Comput Math Methods Med. 2015 Oct 4;2015:462363. doi: 10.1155/2015/462363 (PMC4609422; doi:10.1155/2015/462363)
Supplement: Supplementary file 1 — The Supplementary Material contains five files. In detail, Supplementary Material I lists genes and chemicals related to prostate cancer; Supplementary Material II lists candidate genes and chemicals, their betweenness and p-values; Supplementary Material III lists significant candidate genes and chemicals, their betweenness and p-values; Supplementary Material IV lists KEGG enrichment results of 187 significant candidate genes; Supplementary Material V lists GO enrichment results of 187 significant candidate genes. [file 462363.f1.zip › Supp-I.pdf]

Supplementary Material I. Genes and chemicals related to prostate cancer

1. 309 genes related to prostate cancer

   ACPP
   ADRA1A
   AGR2
   ALCAM
   ALDH1A2
   ALKBH3
   ALPHA
   AMACR
   ANP32C
   ANXA1
   ANXA7
   APA
   APAF1
   APT1
   AR
   ARHGEF26
   ASRGL1
   AXL
   BAGE
   BCLAF1
   BECN1
   BEND4
   BIN1
   BMP10
   BMP-2
   BMP4
   BNIP3L
   BRCA1
   BRCA2
   CACNA1G
   CADM4
   CAND1
   CARM1
   CAV1
   CBS
   CD59
   CDK4
   CDKN1A
   CDKN2A
   CEACAM1
   CEBPA

CELL
CEP192
CERS2
CHEK2
CHK2
CLNS1A
CLU
CMTM5
COL18A1
COX-1
CREB3L4
CRP1
CSTA
CTNNA1
CTNND1
CTPS1
CTTN
CYP24
DAB2
DAB2IP
DAO
DBP
DCC
DKC1
DLC1
DMBT1
DNMT1
DPH1
DSC1
EAF2
EBAG9
EDNRB
EEF1B2
EEF1D
EGFR
EHBP1
EHF
EIF2AK2
EIF4EBP1
EIF5B
ELAC2
EPHA2
EPHB2
FBXO25

FGF10
FGF2
FGF3
FGF8
FLT-1
FOLH1
FOXO1
FOXP3
FRYL
FSHR
FZD1
G3BP1
GAMMA
GAPDH
GAS5
GAS6
GBX1
GBX2
GGNBP2
GGT
GHR
GHRH
GLI2
GLIPR1
GPX1
GPX3
GREB1
GSTT1
H19
H2AFV
HDAC1
HDGF
HIC1
HK2
HLA-A
HMGA2
HN1
HNF1B
HNRNPA3
HNRNPU
HPC2
HSP27
HSP60
HTATIP2

HTR
IGF2R
IGFBP-3
IL24
INHA
INT2
INT-2
IRS-1
ITGA7
JAK1
JAK2
JDP2
JTB
KCNRG
KGF
KLF5
KLF6
KLK10
KLK4
LATS2
LEU
LGALS8
LHR
LIPASE
LITAF
LPL
LRRC26
LZTS1
MAGED2
MAP1B
MAP3K7CL
MAP4
MBP
MCL-1
MCM2
MCM3
MEG3
MFAP1
MGP
MIR1-1
MIR15A
MIR193B
MIR449A
MRP-1

MRP3
MSMB
MSR1
MTHFD1
MX1
MXI1
MYH9
NCL
NCOA4
NDRG1
NF1
NF2
NGFR
NKX3-1
NOP56
NSD1
NUCKS1
OAS3
P120
P21
P450
P51
PARM1
PASP
PAX6
PBOV1
PCAP
PCDH11Y
PCGF2
PCOTH
PDAP1
PDLIM4
PEBP1
PERP
PFKL
PGRMC1
PHB
PHLPP1
PIK3C2G
PIN
PLA2
PLK-1
PML
PPP2CB

PPP3CC
PRAME
PRDM2
PRKAR2A
PSA
PSCA
PSMA
PSMA3
PSPH
PTEN
PTENP1
PTGES3
PTPRJ
PWP1
RAGE
RALY
RASL11A
RASSF1
RB1
RB2
RDH11
RGS3
RNASEL
RND3
RNF113A
RPL10
RPL28
RYK
S100A2
SCFD1
SEC14L2
SERPINB5
SHH
SKP2
SLC39A1
SLC43A1
SLC45A3
SLC5A8
SMAD4
SMAD6
SMAD7
SMN1
SNORD50A
SOX7

SPDEF
SPRY2
SSTR1
SSTR2
SSTR5
ST13
ST7
STAT3
STEAP4
STK39
SUFU
SULF1
TAOK2
TBP
TCHP
TFPI2
TFRC
TGFA
TGFBR1
TIMP-1
TIMP-2
TMEFF2
TMPO
TNF
TNKS1BP1
TOP2B
TP53
TP63
TRH
TRPM8
TRPV1
TRPV6
TSC1
TYK2
UFO
UGT
USP10
USP2
VIP
VWF
WNK1
WNT5A
WT1
WWOX

XPC
YBX1
ZFHX3
ZFPM1

2. 106 chemicals related to prostate cancer
CID000000299
CID000000727
CID000000896
CID000001530
CID000001725
CID000002145
CID000002336
CID000002353
CID000002375
CID000002514
CID000002662
CID000002907
CID000002955
CID000003035
CID000003036
CID000003039
CID000003117
CID000003157
CID000003385
CID000003397
CID000003589
CID000003657
CID000003672
CID000003690
CID000004055
CID000004212
CID000004380
CID000004463
CID000004534
CID000005035
CID000005212
CID000005216
CID000005238
CID000005401
CID000005426
CID000005455
CID000005743
CID000005746
CID000005754

CID000005755
CID000005757
CID000005865
CID000005870
CID000005978
CID000005991
CID000005995
CID000006001
CID000006010
CID000006013
CID000006279
CID000006323
CID000006623
CID000006636
CID000008268
CID000009444
CID000012699
CID000013676
CID000014985
CID000016590
CID000023978
CID000023994
CID000024934
CID000031703
CID000036462
CID000036523
CID000041867
CID000050224
CID000060749
CID000060953
CID000062275
CID000064982
CID000066414
CID000091469
CID000092729
CID000107935
CID000107971
CID000108143
CID000115196
CID000126941
CID000148123
CID000161597
CID000241902
CID000443495

CID000445154
CID000446925
CID000448537
CID000449171
CID000460612
CID001548943
CID002733525
CID003001028
CID003032325
CID005280343
CID005280373
CID005280443
CID005280453
CID005280795
CID005280961
CID005281104
CID005281614
CID005281707
CID005282360
CID005283731
CID005353562
CID005359596
CID011020241
