# Supplementary material for: Identifying New Candidate Genes and Chemicals Related to Prostate Cancer Using a Hybrid Network and Shortest Path Approach
Source: Comput Math Methods Med. 2015 Oct 4;2015:462363. doi: 10.1155/2015/462363 (PMC4609422; doi:10.1155/2015/462363)
Supplement: Supplementary file 1 — The Supplementary Material contains five files. In detail, Supplementary Material I lists genes and chemicals related to prostate cancer; Supplementary Material II lists candidate genes and chemicals, their betweenness and p-values; Supplementary Material III lists significant candidate genes and chemicals, their betweenness and p-values; Supplementary Material IV lists KEGG enrichment results of 187 significant candidate genes; Supplementary Material V lists GO enrichment results of 187 significant candidate genes. [file 462363.f1.zip › Supp-II.pdf]

Supplementary Material II. Candidate genes and chemicals, their betweenness and p-values

Chemical or gene ID	Chemical or gene name	Betweenness	P-value
CID000000051	alpha-ketoglutarate	326	0.844
CID000000177	acetaldehyde	3	0.854
CID000000190	adenine	1	0.987
CID000000222	ammonia	4	0.884
CID000000259	bromide	363	0.002
CID000000271	calcium	9377	0.936
CID000000281	carbon monoxide	342	0.287
CID000000283	formate	344	0.851
CID000000305	choline	725	0.685
CID000000311	citric acid	363	0.186
CID000000312	chloride	2338	0.286
CID000000679	DMSO	76	0.586
CID000000681	dopamine	23	0.967
CID000000712	formalin	361	0.159
CID000000750	glycine	369	0.662
CID000000753	glycerol	1713	0.967
CID000000774	histamine	524	0.834
CID000000784	H2O2	721	0.227
CID000000813	potassium	85	0.578

CID000000888	magnesium	1219	0.991
CID000000903	N-acetylserotonin	3	0.159
CID000000923	sodium	595	0.992
CID000000961	hydroxyl radicals	47	1
CID000000977	oxygen	2648	0.94
CID000000985	palmitate	363	0.601
CID000000991	parathion	362	0.737
CID000001051	pyridoxal 5'-phosphate	364	0.679
CID000001135	thymine	1	0.121
CID000001567	2-mercaptoethanol	4	0.345
CID000001935	tacrine	302	0.126
CID000001983	acetaminophen	1	0.158
CID000002519	caffeine	371	0.028
CID000002520	verapamil	63	0.13
CID000002769	cisapride	4	0.811
CID000002818	clozapine	14	0.699
CID000003032	diclofenac	398	0.065
CID000003715	indomethacin	868	0.084
CID000003776	isopropanol	3	0.056
CID000003779	isoproterenol	1	0.916
CID000004114	8-MOP	2	0.466
CID000004585	olanzapine	2	0.959

CID000004636	oxymetazoline	14	0.001
CID000004763	phenobarbital	22	0.668
CID000004893	prazosin	2	0.09
CID000005202	serotonin	18	0.917
CID000005566	trifluoperazine	363	0.001
CID000005789	thymidine	7	0.129
CID000005815	epinephrine	260	0.05
CID000005833	spironolactone	24	0.079
CID000005885	NADP	1179	0.772
CID000005892	beta-NAD	1234	0.701
CID000005950	L-alanine	17	0.537
CID000005951	L-serine	363	0.074
CID000005957	adenosine triphosphate	14668	1
CID000005960	L-aspartate	363	0.011
CID000005961	glutamine	355	0.205
CID000005962	lysine	23	0.733
CID000005997	cholesterol	359	0.846
CID000006022	adenosine diphosphate	1757	0.145
CID000006031	uridine diphosphate	4	0.979
CID000006076	cyclic AMP	363	0.319
CID000006083	adenosine monophosphate	360	0.71
CID000006106	L-leucine	363	0.057

CID000006134	lactose	17	0.099
CID000006137	L-methionine	1	0.414
CID000006140	phenylalanine	25	0.249
CID000006176	cytidine triphosphate	50	0.119
CID000006322	arginine	210	0.82
CID000006503	tris	1083	0.011
CID000006804	5'-GMP	363	0.129
CID000006830	guanosine triphosphate	1435	0.999
CID000008977	guanosine diphosphate	100	0.998
CID000023925	Fe(III	2089	0.223
CID000023930	manganese	23	0.931
CID000024139	chitin	1	0.962
CID000030323	anthracycline	3	0.124
CID000033032	glutamic acid	1081	0.132
CID000054687	pravastatin	349	0.206
CID000060662	mibefradil	363	0.013
CID000060699	topotecan	174	0.06
CID000060961	adenosine	347	0.641
CID000064689	D-glucose	265	0.736
CID000065036	allicin	2	0.024
CID000065063	dUMP	6	0.479
CID000066370	alpha-D-glucose	1	0.72

CID000072886	L-cysteate	363	0.064
CID000087642	coenzyme A	111	0.987
CID000119400	tartrate	363	0.005
CID000124886	Glutathione	9	0.459
CID000145742	L-proline	4	0.236
CID000157350	hydroxyl radical	362	0.005
CID000161930	icilin	363	0
CID000439155	AdoHcy	11	0.99
CID000439260	norepinephrine	918	0.11
CID000439353	galactose	346	0.38
CID000444795	retinoic acid	374	0.314
CID000444899	arachidonic acid	497	0.477
CID000520535	superoxide	362	0.099
CID000638015	11-cis-retinal	359	0.08
CID000643975	flavin adenine dinucleotide	378	0.299
CID005280360	PGE2	50	0.518
CID005280489	beta-carotene	363	0.173
ENSP00000003084	CFTR	5392	1
ENSP00000005257	RALA	362	0.116
ENSP00000006053	CX3CL1	363	0.001
ENSP00000008527	CRY1	363	0.012
ENSP00000010338	TRAF3IP3	363	0.017

ENSP00000011653	CD4	123	0.721
ENSP00000046794	LCP2	22	0.808
ENSP00000156109	GPKOW	1	0.195
ENSP00000160373	CTTNBP2	86	0.028
ENSP00000162330	BCAR1	111	0.905
ENSP00000162749	TNFRSF1A	1253	0.003
ENSP00000170630	IL4R	7	0.47
ENSP00000173229	NTN1	365	0.042
ENSP00000196551	RPS5	363	0.011
ENSP00000206249	ESR1	11815	0.001
ENSP00000209728	CDC6	186	0.472
ENSP00000212015	SIRT1	92	0.177
ENSP00000212355	TGFBR3	362	0.006
ENSP00000215479	AMELY	363	0
ENSP00000215587	POLR2E	5	0.541
ENSP00000215829	SNRPD3	72	0.977
ENSP00000215832	MAPK1	696	0.309
ENSP00000215909	LGALS1	14	0.172
ENSP00000216117	HMOX1	363	0.072
ENSP00000216223	IL2RB	12	0.357
ENSP00000216225	RBX1	74	0.799
ENSP00000216554	EIF5	363	0.011

ENSP00000216797	NFKBIA	178	0.275
ENSP00000216862	CYP24A1	36	0
ENSP00000216911	AURKA	363	0.095
ENSP00000217244	CSNK2A1	362	0.316
ENSP00000219070	MMP2	363	0.052
ENSP00000219476	TSC2	466	0.069
ENSP00000221494	SF3A2	744	0.695
ENSP00000221930	TGFB1	1003	0.046
ENSP00000221957	PLIN3	363	0.001
ENSP00000222005	CDC37	497	0.108
ENSP00000222254	PIK3R2	5	0.036
ENSP00000223023	WASL	48	0.985
ENSP00000223129	RPA3	191	0.148
ENSP00000223321	PSMA2	251	0.236
ENSP00000224181	C8G	363	0.003
ENSP00000225831	CCL2	363	0.009
ENSP00000225983	HDAC5	1	0.105
ENSP00000226218	VTN, SEBOX	4	0.689
ENSP00000226413	GNRHR	363	0
ENSP00000226574	NFKB1	363	0.011
ENSP00000226730	IL2	19	0.181
ENSP00000227378	HSPA8	224	0.816

ENSP00000227507	CCND1	2792	0.005
ENSP00000227758	BIRC2	1583	0
ENSP00000228307	PXN	112	0.62
ENSP00000228682	GLI1	358	0.041
ENSP00000228837	FGF6	1074	0.003
ENSP00000228872	CDKN1B	54	0.71
ENSP00000228916	SCNN1A	258	0.184
ENSP00000229022	VDR	1147	0.02
ENSP00000229769	FANCE	3	0.356
ENSP00000229794	MAPK14	748	0.031
ENSP00000230449	EXOC2	363	0.058
ENSP00000231487	SKP1	191	0.651
ENSP00000231509	NR3C1	2957	0.001
ENSP00000232014	BCL6	3	0.211
ENSP00000233242	APOB	5	0.407
ENSP00000233946	IL1R1	1	0.336
ENSP00000234111	ODC1	1	0.928
ENSP00000234310	PPP3R1	363	0.003
ENSP00000235090	WDR77	702	0.295
ENSP00000236850	APOA1	6	0.837
ENSP00000237612	ABCG2	376	0.048
ENSP00000239223	DUSP1	119	0.132

ENSP00000240185	TARDBP	10	0.996
ENSP00000241337	GSTM2	2	0.037
ENSP00000242057	AHR	361	0.585
ENSP00000242577	DYNLL1	363	0.355
ENSP00000244007	PLCG1	2110	0
ENSP00000245323	EFNB2	364	0.01
ENSP00000245457	PTGER2	4	0.049
ENSP00000245541	GGA3	564	0.022
ENSP00000245544	NUP85	362	0.011
ENSP00000245960	CDC25B	63	0.516
ENSP00000247161	ELK1	121	0.093
ENSP00000247668	TRAF2	1898	0.01
ENSP00000248553	HSPB1	363	0.048
ENSP00000248566	SHFM1	45	0.979
ENSP00000249071	RAC2	102	0.652
ENSP00000249299	NAA38	17	0.498
ENSP00000250495	NEDD8	433	0.054
ENSP00000251810	RRM2B	343	0.032
ENSP00000251849	RAF1	2652	0.35
ENSP00000251968	TSG101	363	0.218
ENSP00000252029	TYMP	9	0.097
ENSP00000252506	GADD45G	8	0.01

ENSP00000252622	LSM7	2	1
ENSP00000252945	CYP2E1	4	0.615
ENSP00000252997	GATA5	40	0.049
ENSP00000254066	RARA	1218	0.042
ENSP00000254122	FSHB	363	0.03
ENSP00000254227	NR0B2	363	0.106
ENSP00000254301	LGALS3	3	0.062
ENSP00000254657	PER2	363	0.023
ENSP00000254719	RPA1	191	0.252
ENSP00000254942	TERF2	277	0.397
ENSP00000256216	HSD17B4	363	0.003
ENSP00000256383	EIF2S1	359	0.236
ENSP00000256442	CCNB1	2418	0.469
ENSP00000256474	VHL	786	0.741
ENSP00000256857	GRP	2	0.612
ENSP00000256958	SLCO1B1	357	0.195
ENSP00000256996	DDB2	13	0.222
ENSP00000258418	CAB39	362	0.021
ENSP00000258743	IL6	361	0.158
ENSP00000258962	SRSF1	16	0.721
ENSP00000259808	RIPK1	328	0.294
ENSP00000260010	TLR2	1	0.14

ENSP00000260433	CYP19A1	1426	0.011
ENSP00000260682	CYP2C9	363	0.119
ENSP00000260762	EXOC6	1	0.325
ENSP00000261023	ITGAV	3	0.801
ENSP00000261349	LRP6	1086	0.011
ENSP00000261366	LMNB1	363	0.048
ENSP00000261461	PPP2R5A	86	0.355
ENSP00000261733	ALDH2	363	0.017
ENSP00000261769	CDH1	364	0.164
ENSP00000261799	PDGFRB	844	0.003
ENSP00000261908	NEO1	4	0.067
ENSP00000262053	ATF1	362	0.002
ENSP00000262160	SMAD2	842	0.118
ENSP00000262209	TRPA1	363	0.022
ENSP00000262238	YY1	363	0.15
ENSP00000262320	AXIN1	1874	0.031
ENSP00000262367	CREBBP	3569	0
ENSP00000262435	SMURF2	15	0.067
ENSP00000262477	RABEP1	363	0.189
ENSP00000262613	SLC9A3R1	118	0.811
ENSP00000262629	TYROBP	316	0.599
ENSP00000262643	CCNE1	110	0.717

ENSP00000262768	TIMP2	9	0.211
ENSP00000262803	UPF1	8	0.882
ENSP00000262809	ELL	363	0
ENSP00000263025	MAPK3	357	0.024
ENSP00000263125	PRKCQ	363	0.001
ENSP00000263126	AKR1C4	363	0
ENSP00000263253	EP300	1743	0.315
ENSP00000263277	EHD2	363	0
ENSP00000263408	C9	363	0
ENSP00000263734	EPAS1	3	0.802
ENSP00000263753	SGOL1	221	0.817
ENSP00000263915	GRB14	1	0.001
ENSP00000263918	STRN	10	0.049
ENSP00000263923	KDR	2	0.305
ENSP00000263946	PKP1	1	0
ENSP00000263967	PIK3CA	583	0.911
ENSP00000264001	CKLF	363	0
ENSP00000264033	CBL	3418	0.624
ENSP00000264110	ATF2	459	0.047
ENSP00000264156	MCM6	6	0.097
ENSP00000264246	CD80	102	0.306
ENSP00000264381	BCHE	738	0.651

ENSP00000264708	POMC	705	0.275
ENSP00000264832	ICAM1	363	0.035
ENSP00000264998	TF	2	0.628
ENSP00000265171	EGF	82	0.322
ENSP00000265335	RAD50	266	0.433
ENSP00000265354	SRF	719	0.069
ENSP00000265717	PRKAR2B	363	0.081
ENSP00000265724	ABCB1	389	0.077
ENSP00000265734	CDK6	363	0.014
ENSP00000265965	SERGEF	363	0
ENSP00000266000	DAXX	7	0.381
ENSP00000266970	CDK2	813	0.627
ENSP00000267082	ITGB7	233	0.027
ENSP00000267101	ERBB3	6	0.486
ENSP00000267415	TINF2	277	0.159
ENSP00000267430	FANCM	3	0.235
ENSP00000267868	RAD51	329	0.322
ENSP00000268035	IGF1R	180	0.869
ENSP00000268182	IQGAP1	1	0.983
ENSP00000268712	NCOR1	216	0.252
ENSP00000269141	CDH2	2217	0.023
ENSP00000269260	ARRB2	360	0.41

ENSP00000269321	ARHGDIA	163	0.44
ENSP00000269349	EIF4A3	12	0.852
ENSP00000269397	CBX4	362	0.166
ENSP00000269571	ERBB2	882	0.284
ENSP00000270202	AKT1	3084	0.205
ENSP00000271628	SF3B4	5	0.307
ENSP00000272190	REN	804	0.029
ENSP00000272298	CALM2	358	0.031
ENSP00000273047	RAB5A	363	0.037
ENSP00000274026	CCNA2	842	0.867
ENSP00000274335	PIK3R1	249	0.965
ENSP00000274376	RASA1	856	0.006
ENSP00000276198	HTR2C	8	0.624
ENSP00000276201	UPF3B	9	0.983
ENSP00000276414	GNRH1	363	0.031
ENSP00000276431	TNFRSF10B	11	0
ENSP00000276571	CRH	360	0.256
ENSP00000276603	TERF1	363	0.016
ENSP00000277541	NOTCH1	116	0.875
ENSP00000278568	PAK1	1	0.993
ENSP00000278616	ATM	370	0.538
ENSP00000278916	CHEK1	11	0.626

ENSP00000279488	DUSP6	9	0.029
ENSP00000280097	HNMT	284	0.188
ENSP00000280155	ADRA2A	4	0.29
ENSP00000280193	VEGFC	1	0.057
ENSP00000280892	EIF4E	720	0.182
ENSP00000281623	FBXO4	86	0.031
ENSP00000281708	FBXW7	13	0.838
ENSP00000281821	EPHA4	2	0.055
ENSP00000282091	PTH	327	0.211
ENSP00000282441	YAP1	362	0.113
ENSP00000282561	GJA1	20	0.673
ENSP00000283635	CD8A	13	0.047
ENSP00000284384	PRKCA	230	0.127
ENSP00000284523	WNT3A	725	0.007
ENSP00000284811	TCEB1	1	0.802
ENSP00000284957	RABGEF1	359	0.233
ENSP00000284981	APP	361	0.51
ENSP00000286627	KCNMA1	363	0.009
ENSP00000286648	DCK	88	0.104
ENSP00000287598	BUB1B	562	0.682
ENSP00000287641	SST	1085	0
ENSP00000287647	FANCD2	5	0.613

ENSP00000287727	ZFYVE9	362	0.139
ENSP00000287820	PPARG	808	0.419
ENSP00000287936	HMGCR	706	0.84
ENSP00000288422	TAB3	363	0
ENSP00000288986	NCK1	1	0.993
ENSP00000290330	SNF8	363	0.001
ENSP00000290921	CTBP1	362	0.289
ENSP00000291552	U2AF1	7	0.824
ENSP00000292303	CCR5	123	0.409
ENSP00000292644	PSMC2	326	0.994
ENSP00000293288	BAX	180	0.149
ENSP00000293308	KRT8	363	0
ENSP00000293379	ITGA5	649	0.308
ENSP00000293549	WNT1	363	0.006
ENSP00000294172	NXF1	362	0.52
ENSP00000294304	LRP5	363	0.004
ENSP00000294954	LHCGR	363	0
ENSP00000295408	MERTK	724	0
ENSP00000295600	MITF	363	0
ENSP00000295731	IHH	3	0.059
ENSP00000295897	ALB	66	0.779
ENSP00000296181	ITGB5	1	0.037

ENSP00000296575	HHIP	3	0.026
ENSP00000296581	LSM6	4	0.149
ENSP00000296585	ITGA2	36	0.749
ENSP00000296930	NPM1	1082	0.083
ENSP00000297494	NOS3	419	0.599
ENSP00000298139	WRN	11	0.412
ENSP00000298316	ARF6	1	0.61
ENSP00000298772	TRIM13	363	0
ENSP00000299402	APBB1	150	0.625
ENSP00000299421	ILK	15	0.454
ENSP00000299543	CTDP1	4	0.966
ENSP00000300093	PLK1	242	0.961
ENSP00000300134	STAT6	382	0.077
ENSP00000300161	YWHAB	194	0.318
ENSP00000300413	SNRPD1	363	0.09
ENSP00000300574	CRK	227	0.338
ENSP00000300651	MED1	17	0.957
ENSP00000300738	RRM1	363	0.008
ENSP00000300935	RAB8A	1	0.273
ENSP00000301019	CDT1	11	0.459
ENSP00000301141	CYP2A6	2	0.592
ENSP00000301633	BIRC5	7	0.648

ENSP00000301764	DDB1	13	0.555
ENSP00000301838	FADD	5	0.311
ENSP00000302150	PRL	4	0.3
ENSP00000302486	MAP2K1	623	0.197
ENSP00000302530	BUB1	169	0.927
ENSP00000302564	BCL2L1	377	0.077
ENSP00000302665	IGF1	6	0.926
ENSP00000302811	MTNR1A	4	0.146
ENSP00000302955	RRM2	65	0.085
ENSP00000302967	HDAC3	13	0.643
ENSP00000303242	ITGB2	361	0.221
ENSP00000303706	CDC25A	3	0.293
ENSP00000303830	INSR	252	0.918
ENSP00000303939	CTLA4	307	0.286
ENSP00000304236	CD14	1	0.028
ENSP00000304895	IRS1	429	0.748
ENSP00000305372	ADRB2	915	0.5
ENSP00000305769	SMAD1	5	0.08
ENSP00000306245	FOS	911	0.606
ENSP00000306512	IL8	363	0.135
ENSP00000307235	EIF2AK3	345	0.162
ENSP00000307288	MCM7	42	0.247

ENSP00000307863	U2AF2	645	0.814
ENSP00000308450	CDC20	205	0.707
ENSP00000308741	CLOCK	363	0.013
ENSP00000308938	PLG	3	0.899
ENSP00000309103	BAD	135	0.097
ENSP00000309503	YWHAZ	325	0.461
ENSP00000309572	TERT	373	0.013
ENSP00000309845	HRAS	4247	0.455
ENSP00000310127	IRF3	748	0.003
ENSP00000310596	LSM1	11	0.759
ENSP00000311032	CASP3	2098	0.001
ENSP00000311113	JUP	423	0.102
ENSP00000311430	RPL4	1	0.088
ENSP00000311469	GSTM1	7	0.124
ENSP00000311502	HEG1	362	0.003
ENSP00000311579	TNKS	363	0
ENSP00000311677	PPP1R8	1	0.67
ENSP00000312652	LEP	220	0.514
ENSP00000312735	POLR2B	9	0.919
ENSP00000312987	HNF4A	1	0.035
ENSP00000312995	CLSPN	4	0.69
ENSP00000313199	HNRNPD	333	0.159

ENSP00000313420	PRKDC	356	0.408
ENSP00000313829	KHDRBS1	1090	0.767
ENSP00000314004	ANAPC2	5	0.503
ENSP00000314458	CDC42	74	1
ENSP00000314491	SRRT	224	0.349
ENSP00000314949	POLR2A	31	0.888
ENSP00000315615	AKAP5	363	0.068
ENSP00000315644	TYMS	1056	0.05
ENSP00000315702	MOB4	217	0.138
ENSP00000315859	RNPS1	2	0.844
ENSP00000315997	LILRB1	363	0
ENSP00000316136	KCNJ1	362	0.009
ENSP00000316152	SFTPC	1	0.019
ENSP00000316460	FYB	7	0.201
ENSP00000316786	HSD11B2	363	0.001
ENSP00000316879	EIF4G1	725	0.121
ENSP00000318472	NCAM1	2	0.21
ENSP00000318861	SF3B2	2	0.8
ENSP00000319060	CAMK2G	362	0.025
ENSP00000319169	PRMT5	356	0.216
ENSP00000319788	NQO1	1	0.042
ENSP00000320147	EZH2	352	0.278

ENSP00000320180	GHRHR	358	0.03
ENSP00000320940	NCOA1	3886	0
ENSP00000321656	CDC25C	17	0.185
ENSP00000323050	RBBP8	93	0.189
ENSP00000324648	CYP2B6	363	0.155
ENSP00000324729	SAV1	2	0.203
ENSP00000324804	PPP2R1A	86	0.222
ENSP00000324806	GSK3B	317	0.565
ENSP00000324856	STK11	362	0.144
ENSP00000324890	CD28	102	0.391
ENSP00000324897	UBE2I	415	0.817
ENSP00000326031	PPP1CA	1	0.631
ENSP00000326366	PSEN1	167	0.502
ENSP00000326804	CUL1	371	0.121
ENSP00000327246	VIPR1	361	0.008
ENSP00000327336	BGN	363	0
ENSP00000329357	SP1	220	0.545
ENSP00000329380	GP1BA	267	0.383
ENSP00000329411	IRF7	2	0.412
ENSP00000329623	BCL2	515	0.206
ENSP00000329967	TBK1	19	0.338
ENSP00000330054	EEF1A1	724	0.001

ENSP00000330237	CASP9	12	0.191
ENSP00000330382	PDGFB	363	0
ENSP00000330393	LEPR	1	0.407
ENSP00000331201	HGS	363	0.021
ENSP00000331358	GAST	220	0.083
ENSP00000332353	PTCH1	1071	0.034
ENSP00000332643	NDN	80	0.034
ENSP00000332973	SMAD3	382	0.114
ENSP00000333001	RBM8A	4	0.646
ENSP00000334458	GATA4	359	0.2
ENSP00000334940	GGN	363	0
ENSP00000335153	HSP90AA1	10574	0.193
ENSP00000335620	GSTA1	2	0.394
ENSP00000336630	ADORA2A	347	0.095
ENSP00000336741	DHX15	4	0.319
ENSP00000336790	ATF4	345	0.234
ENSP00000337014	HFE2	4	0.046
ENSP00000337773	NQO2	2	0.008
ENSP00000337825	LCK	168	0.839
ENSP00000337915	CYP3A4	1453	0.538
ENSP00000338018	HIF1A	1713	0.819
ENSP00000338799	IL6ST	63	0.424

ENSP00000338934	EZR	119	0.77
ENSP00000338983	MUC1	363	0.16
ENSP00000339007	GRB2	999	0.975
ENSP00000339109	ANAPC1	461	0.175
ENSP00000339151	IKBKB	43	0.027
ENSP00000339328	PLAUR	7	0.24
ENSP00000339428	SOCS2	1	0.073
ENSP00000340330	KAT5	152	0.806
ENSP00000340684	MAOA	363	0.039
ENSP00000340858	B2M	2564	0
ENSP00000340944	PTPN11	130	0.486
ENSP00000341189	PTK2	1138	0.053
ENSP00000341268	TRADD	24	0.063
ENSP00000341344	GGA1	4	0.463
ENSP00000341835	MYOCD	1	0.029
ENSP00000342007	CYP1A2	725	0.07
ENSP00000342215	KIR2DL3	12	0.492
ENSP00000342374	SNRPD2	20	0.372
ENSP00000342793	PLD1	231	0.061
ENSP00000342952	ADCY2	61	0.81
ENSP00000343054	RBM5	9	0.653
ENSP00000343925	ESR2	722	0.057

ENSP00000344115	CDH5	1	0.362
ENSP00000344220	PDPK1	18	0.101
ENSP00000344352	ATF3	707	0.06
ENSP00000344456	CTNNB1	4476	0.009
ENSP00000344668	KRIT1	362	0.016
ENSP00000344818	UBC	24398	0.049
ENSP00000345344	CTSL	363	0.043
ENSP00000345530	NEDD4	363	0.003
ENSP00000345571	E2F1	441	0.126
ENSP00000345681	GATA2	2	0.062
ENSP00000345751	SCNN1B	1	0.001
ENSP00000346001	RPL3	1	0.089
ENSP00000346022	RPL9	1	0.695
ENSP00000346294	S100A4	365	0
ENSP00000346839	FN1	833	0.615
ENSP00000347046	PDE5A	363	0.003
ENSP00000347858	XIAP	10	0.306
ENSP00000348307	SIRPA	307	0.118
ENSP00000348461	RAC1	33	0.925
ENSP00000348551	NCOR2	14	0.798
ENSP00000348554	CDC16	16	0.307
ENSP00000348577	RANGAP1	346	0.699

ENSP00000348708	UPF2	11	0.994
ENSP00000348775	ACOX3	363	0
ENSP00000348786	RAP1A	8	0.184
ENSP00000348986	INS-IGF2	251	0.94
ENSP00000349049	KDM1A	1	0.313
ENSP00000349393	LIG4	1	0.025
ENSP00000349467	CALM1	405	0.264
ENSP00000350708	RAD23B	350	0.37
ENSP00000350877	SRSF2	7	0.626
ENSP00000350941	SRC	2389	0.136
ENSP00000351273	CASP8	30	0.452
ENSP00000351407	ARNT	336	0.667
ENSP00000351486	NTRK1	1001	0.021
ENSP00000351490	MAX	363	0.022
ENSP00000351908	MAP3K5	371	0.047
ENSP00000351997	MAP2K6	508	0.002
ENSP00000352400	NUP214	352	0.434
ENSP00000352608	RYR1	22	0.383
ENSP00000352842	PFKM	363	0.02
ENSP00000352929	CSNK1E	363	0.039
ENSP00000352980	HIST1H4A	28	0.667
ENSP00000353483	MAPK8	195	0.688

ENSP00000353915	HTR4	4	0.772
ENSP00000354394	STAT1	591	0.263
ENSP00000354458	C8A	363	0
ENSP00000354511	COMT	1	0.267
ENSP00000354522	TOP1	174	0.301
ENSP00000354558	MTOR	552	0.529
ENSP00000354609	CNKSR1	363	0
ENSP00000354612	PTGS1	591	0.053
ENSP00000354621	SMURF1	362	0.046
ENSP00000354859	DRD2	2	0.837
ENSP00000354927	MAP3K3	362	0.048
ENSP00000355261	SMG5	8	0.242
ENSP00000355759	PARP1	355	0.455
ENSP00000355930	SLC22A1	1	0.008
ENSP00000356070	MAPKAPK2	369	0.052
ENSP00000356087	IKBKE	29	0.233
ENSP00000356236	SYT2	1	0
ENSP00000356438	PTGS2	719	0.318
ENSP00000356480	RNF2	1	0.141
ENSP00000356541	SF3B5	4	0.076
ENSP00000356832	SGK1	277	0.014
ENSP00000357392	EFNA1	2	0.1

ENSP00000357656	FYN	217	0.936
ENSP00000357674	SNAPIN	1	0.084
ENSP00000357858	BUB3	690	0.37
ENSP00000357879	PSMD4	350	0.496
ENSP00000358022	MCL1	397	0.242
ENSP00000358335	MAP3K7	477	0.011
ENSP00000358525	NGF	950	0.14
ENSP00000358541	SIKE1	29	0.248
ENSP00000358595	CGA	364	0.049
ENSP00000358622	IKBKG	355	0.321
ENSP00000358727	GSTO1	5	0.104
ENSP00000358866	FLNA	363	0.024
ENSP00000359206	BTRC	44	0.276
ENSP00000359211	DPYD	362	0.028
ENSP00000359345	RPL5	361	0.585
ENSP00000359531	GTF2B	12	0.901
ENSP00000359998	GSTA4	4	0.206
ENSP00000360141	GNAS	1	0.071
ENSP00000360266	JUN	2673	0.123
ENSP00000360683	PTPN1	403	0.16
ENSP00000360869	IFIT1	362	0.049
ENSP00000361066	NCOA3	77	0.127

ENSP00000361120	RALGDS	363	0
ENSP00000361125	VEGFA	346	0.52
ENSP00000361366	SFTPD	363	0
ENSP00000361418	IPO13	84	0.41
ENSP00000361423	ABL1	48	0.949
ENSP00000361512	PRPS1	360	0.016
ENSP00000361892	STK4	2	0.192
ENSP00000362413	PGK1	353	0.072
ENSP00000362820	SRSF3	7	0.541
ENSP00000363055	ZWINT	363	0.246
ENSP00000363081	DKK1	5	0.112
ENSP00000363377	FOXO4	49	0.009
ENSP00000363512	ALOX5	366	0.04
ENSP00000363591	BAK1	4	0.005
ENSP00000363641	TXN	9	0.21
ENSP00000363708	BMPR2	4	0.166
ENSP00000363832	AOX1	271	0.025
ENSP00000364094	ITGB1	718	0.348
ENSP00000364403	UBR4	363	0
ENSP00000364847	MAGED1	363	0.001
ENSP00000364898	SYK	316	0.695
ENSP00000365435	TNFRSF1B	363	0.002

ENSP00000365439	HNRNPK	960	0.823
ENSP00000365858	GATA1	2	0.025
ENSP00000365891	WAS	24	0.956
ENSP00000366135	EXOSC10	10	0.995
ENSP00000366565	VPS28	363	0.019
ENSP00000367207	MYC	729	0.25
ENSP00000367299	VPS36	363	0.003
ENSP00000367309	MAOB	1	0.351
ENSP00000367316	ITGA8	284	0.172
ENSP00000367910	FANCG	360	0.077
ENSP00000367959	HTR2A	4	0.806
ENSP00000368104	BMP2	442	0.218
ENSP00000368438	PCNA	1826	0.612
ENSP00000368683	EDN1	364	0.051
ENSP00000368699	ISG15	724	0.033
ENSP00000368766	ADRA1D	262	0.017
ENSP00000369050	CYP1A1	6	0.691
ENSP00000369213	DDX58	724	0.033
ENSP00000369816	SHBG	363	0.01
ENSP00000370343	IRF4	3	0.152
ENSP00000370473	IGFBP3	9	0.894
ENSP00000370503	CCM2	362	0.018

ENSP00000371138	FKBP1A	26	0.389
ENSP00000371432	PRLR	4	0.555
ENSP00000371973	SAP18	2	0.266
ENSP00000372793	LTA	160	0.079
ENSP00000372975	HLA-C	8	0.23
ENSP00000376076	SUMO1	15	0.933
ENSP00000376776	DBH	366	0.009
ENSP00000378165	ZNF207	690	0.322
ENSP00000378323	PPP3CA	363	0.05
ENSP00000378529	FZR1	21	0.333
ENSP00000379213	PTHLH	3	0.112
ENSP00000379330	NFATC2	37	0.082
ENSP00000379625	MYD88	2	0.552
ENSP00000380150	CARD11	355	0.059
ENSP00000380227	ITGA4	258	0.145
ENSP00000380280	FGFR1	1798	0.001
ENSP00000381331	HDAC2	354	0.513
ENSP00000381607	GSTP1	843	0.001
ENSP00000382166	CX3CR1	363	0
ENSP00000382342	ABCC1	350	0.1
ENSP00000382697	ROCK1	363	0.001
ENSP00000382723	AGPAT1	1	0.058

ENSP00000383199	NEDD4L	259	0.151
ENSP00000384053	CSF2RB	3	0.531
ENSP00000384273	RELA	732	0.209
ENSP00000384675	SOS1	477	0.914
ENSP00000385021	FANCL	363	0
ENSP00000386069	ADRA2C	10	0.281
ENSP00000386171	ESRRG	363	0.094
ENSP00000386717	RPL31	1	0.005
ENSP00000386896	ITGA6	3	0.252
ENSP00000387286	RAB1A	348	0.264
ENSP00000387662	GCG	220	0.375
ENSP00000389934	EXOC5	1	0.364
ENSP00000390500	STK3	2	0.235
ENSP00000391592	PTPN6	3	0.529
ENSP00000396127	RAN	350	0.693
ENSP00000396308	DHFR	369	0.152
ENSP00000396439	RING1	362	0.019
ENSP00000399968	NCOA2	14	0.063
ENSP00000400175	RHOA	363	0.099
ENSP00000401303	SHC1	936	0.044
ENSP00000401802	PSMC6	112	0.135
ENSP00000401980	MAVS	2	0.359

ENSP00000404121	ILF3	366	0.152
ENSP00000407431	HLA-C	12	0.44
ENSP00000409132	HLA-G	414	0.134
ENSP00000410294	FGFR2	1085	0
ENSP00000411532	TOP2A	360	0.144
ENSP00000411698	USO1	363	0.079
ENSP00000412045	TXNRD1	1	0.029
ENSP00000413720	CDKN1C	363	0
ENSP00000414634	LSM2	9	0.967
ENSP00000415183	MUC2	363	0.002
ENSP00000415615	CSNK2B	362	0.037
ENSP00000416097	GOLGA2	15	0.198
ENSP00000417281	MDM2	420	0.972
ENSP00000417404	HFE	794	0.015
ENSP00000418447	PPP2CA	244	0.812
ENSP00000419599	DPH3	363	0
ENSP00000419692	RXRA	52	0.869
ENSP00000420168	GSTA2	20	0
