# Supplementary material for: Identifying New Candidate Genes and Chemicals Related to Prostate Cancer Using a Hybrid Network and Shortest Path Approach
Source: Comput Math Methods Med. 2015 Oct 4;2015:462363. doi: 10.1155/2015/462363 (PMC4609422; doi:10.1155/2015/462363)
Supplement: Supplementary file 1 — The Supplementary Material contains five files. In detail, Supplementary Material I lists genes and chemicals related to prostate cancer; Supplementary Material II lists candidate genes and chemicals, their betweenness and p-values; Supplementary Material III lists significant candidate genes and chemicals, their betweenness and p-values; Supplementary Material IV lists KEGG enrichment results of 187 significant candidate genes; Supplementary Material V lists GO enrichment results of 187 significant candidate genes. [file 462363.f1.zip › Supp-III.pdf]

Supplementary Material III. Significant candidate genes and chemicals, their betweenness and p-values

Chemical or gene ID	Chemical or gene name	Betweenness	P-value
CID000000259	bromide	363	0.002
CID000002519	caffeine	371	0.028
CID000004636	oxymetazoline	14	0.001
CID000005566	trifluoperazine	363	0.001
CID000005960	L-aspartate	363	0.011
CID000006503	tris	1083	0.011
CID000060662	mibefradil	363	0.013
CID000065036	allicin	2	0.024
CID000119400	tartrate	363	0.005
CID000157350	hydroxyl radical	362	0.005
CID000161930	icilin	363	0
ENSP00000006053	CX3CL1	363	0.001
ENSP00000008527	CRY1	363	0.012
ENSP00000010338	TRAF3IP3	363	0.017
ENSP00000160373	CTTNBP2	86	0.028
ENSP00000162749	TNFRSF1A	1253	0.003
ENSP00000173229	NTN1	365	0.042
ENSP00000196551	RPS5	363	0.011
ENSP00000206249	ESR1	11815	0.001
ENSP00000212355	TGFBR3	362	0.006
ENSP00000215479	AMELY	363	0
ENSP00000216554	EIF5	363	0.011
ENSP00000216862	CYP24A1	36	0
ENSP00000221930	TGFB1	1003	0.046
ENSP00000221957	PLIN3	363	0.001
ENSP00000222254	PIK3R2	5	0.036
ENSP00000224181	C8G	363	0.003
ENSP00000225831	CCL2	363	0.009
ENSP00000226413	GNRHR	363	0

ENSP00000226574	NFKB1	363	0.011
ENSP00000227507	CCND1	2792	0.005
ENSP00000227758	BIRC2	1583	0
ENSP00000228682	GLI1	358	0.041
ENSP00000228837	FGF6	1074	0.003
ENSP00000229022	VDR	1147	0.02
ENSP00000229794	MAPK14	748	0.031
ENSP00000231509	NR3C1	2957	0.001
ENSP00000234310	PPP3R1	363	0.003
ENSP00000237612	ABCG2	376	0.048
ENSP00000241337	GSTM2	2	0.037
ENSP00000244007	PLCG1	2110	0
ENSP00000245323	EFNB2	364	0.01
ENSP00000245457	PTGER2	4	0.049
ENSP00000245541	GGA3	564	0.022
ENSP00000245544	NUP85	362	0.011
ENSP00000247668	TRAF2	1898	0.01
ENSP00000248553	HSPB1	363	0.048
ENSP00000251810	RRM2B	343	0.032
ENSP00000252506	GADD45G	8	0.01
ENSP00000252997	GATA5	40	0.049
ENSP00000254066	RARA	1218	0.042
ENSP00000254122	FSHB	363	0.03
ENSP00000254657	PER2	363	0.023
ENSP00000256216	HSD17B4	363	0.003
ENSP00000258418	CAB39	362	0.021
ENSP00000260433	CYP19A1	1426	0.011
ENSP00000261349	LRP6	1086	0.011
ENSP00000261366	LMNB1	363	0.048
ENSP00000261733	ALDH2	363	0.017
ENSP00000261799	PDGFRB	844	0.003
ENSP00000262053	ATF1	362	0.002
ENSP00000262209	TRPA1	363	0.022

ENSP00000262320	AXIN1	1874	0.031
ENSP00000262367	CREBBP	3569	0
ENSP00000262809	ELL	363	0
ENSP00000263025	MAPK3	357	0.024
ENSP00000263125	PRKCQ	363	0.001
ENSP00000263126	AKR1C4	363	0
ENSP00000263277	EHD2	363	0
ENSP00000263408	C9	363	0
ENSP00000263915	GRB14	1	0.001
ENSP00000263918	STRN	10	0.049
ENSP00000263946	PKP1	1	0
ENSP00000264001	CKLF	363	0
ENSP00000264110	ATF2	459	0.047
ENSP00000264832	ICAM1	363	0.035
ENSP00000265734	CDK6	363	0.014
ENSP00000265965	SERGEF	363	0
ENSP00000267082	ITGB7	233	0.027
ENSP00000269141	CDH2	2217	0.023
ENSP00000272190	REN	804	0.029
ENSP00000272298	CALM2	358	0.031
ENSP00000273047	RAB5A	363	0.037
ENSP00000274376	RASA1	856	0.006
ENSP00000276414	GNRH1	363	0.031
ENSP00000276431	TNFRSF10B	11	0
ENSP00000276603	TERF1	363	0.016
ENSP00000279488	DUSP6	9	0.029
ENSP00000281623	FBXO4	86	0.031
ENSP00000283635	CD8A	13	0.047
ENSP00000284523	WNT3A	725	0.007
ENSP00000286627	KCNMA1	363	0.009
ENSP00000287641	SST	1085	0
ENSP00000288422	TAB3	363	0
ENSP00000290330	SNF8	363	0.001

ENSP00000293308	KRT8	363	0
ENSP00000293549	WNT1	363	0.006
ENSP00000294304	LRP5	363	0.004
ENSP00000294954	LHCGR	363	0
ENSP00000295408	MERTK	724	0
ENSP00000295600	MITF	363	0
ENSP00000296181	ITGB5	1	0.037
ENSP00000296575	HHIP	3	0.026
ENSP00000298772	TRIM13	363	0
ENSP00000300738	RRM1	363	0.008
ENSP00000304236	CD14	1	0.028
ENSP00000308741	CLOCK	363	0.013
ENSP00000309572	TERT	373	0.013
ENSP00000310127	IRF3	748	0.003
ENSP00000311032	CASP3	2098	0.001
ENSP00000311502	HEG1	362	0.003
ENSP00000311579	TNKS	363	0
ENSP00000312987	HNF4A	1	0.035
ENSP00000315997	LILRB1	363	0
ENSP00000316136	KCNJ1	362	0.009
ENSP00000316152	SFTPC	1	0.019
ENSP00000316786	HSD11B2	363	0.001
ENSP00000319060	CAMK2G	362	0.025
ENSP00000319788	NQO1	1	0.042
ENSP00000320180	GHRHR	358	0.03
ENSP00000320940	NCOA1	3886	0
ENSP00000327246	VIPR1	361	0.008
ENSP00000327336	BGN	363	0
ENSP00000330054	EEF1A1	724	0.001
ENSP00000330382	PDGFB	363	0
ENSP00000331201	HGS	363	0.021
ENSP00000332353	PTCH1	1071	0.034
ENSP00000332643	NDN	80	0.034

ENSP00000334940	GGN	363	0
ENSP00000337014	HFE2	4	0.046
ENSP00000337773	NQO2	2	0.008
ENSP00000339151	IKBKB	43	0.027
ENSP00000340684	MAOA	363	0.039
ENSP00000340858	B2M	2564	0
ENSP00000341835	MYOCD	1	0.029
ENSP00000344456	CTNNB1	4476	0.009
ENSP00000344668	KRIT1	362	0.016
ENSP00000344818	UBC	24398	0.049
ENSP00000345344	CTSL	363	0.043
ENSP00000345530	NEDD4	363	0.003
ENSP00000345751	SCNN1B	1	0.001
ENSP00000346294	S100A4	365	0
ENSP00000347046	PDE5A	363	0.003
ENSP00000348775	ACOX3	363	0
ENSP00000349393	LIG4	1	0.025
ENSP00000351486	NTRK1	1001	0.021
ENSP00000351490	MAX	363	0.022
ENSP00000351908	MAP3K5	371	0.047
ENSP00000351997	MAP2K6	508	0.002
ENSP00000352842	PFKM	363	0.02
ENSP00000352929	CSNK1E	363	0.039
ENSP00000354458	C8A	363	0
ENSP00000354609	CNKSR1	363	0
ENSP00000354621	SMURF1	362	0.046
ENSP00000354927	MAP3K3	362	0.048
ENSP00000355930	SLC22A1	1	0.008
ENSP00000356236	SYT2	1	0
ENSP00000356832	SGK1	277	0.014
ENSP00000358335	MAP3K7	477	0.011
ENSP00000358595	CGA	364	0.049
ENSP00000358866	FLNA	363	0.024

ENSP00000359211	DPYD	362	0.028
ENSP00000360869	IFIT1	362	0.049
ENSP00000361120	RALGDS	363	0
ENSP00000361366	SFTPD	363	0
ENSP00000361512	PRPS1	360	0.016
ENSP00000363377	FOXO4	49	0.009
ENSP00000363512	ALOX5	366	0.04
ENSP00000363591	BAK1	4	0.005
ENSP00000363832	AOX1	271	0.025
ENSP00000364403	UBR4	363	0
ENSP00000364847	MAGED1	363	0.001
ENSP00000365435	TNFRSF1B	363	0.002
ENSP00000365858	GATA1	2	0.025
ENSP00000366565	VPS28	363	0.019
ENSP00000367299	VPS36	363	0.003
ENSP00000368699	ISG15	724	0.033
ENSP00000368766	ADRA1D	262	0.017
ENSP00000369213	DDX58	724	0.033
ENSP00000369816	SHBG	363	0.01
ENSP00000370503	CCM2	362	0.018
ENSP00000376776	DBH	366	0.009
ENSP00000380280	FGFR1	1798	0.001
ENSP00000381607	GSTP1	843	0.001
ENSP00000382166	CX3CR1	363	0
ENSP00000382697	ROCK1	363	0.001
ENSP00000385021	FANCL	363	0
ENSP00000386717	RPL31	1	0.005
ENSP00000396439	RING1	362	0.019
ENSP00000401303	SHC1	936	0.044
ENSP00000410294	FGFR2	1085	0
ENSP00000412045	TXNRD1	1	0.029
ENSP00000413720	CDKN1C	363	0
ENSP00000415183	MUC2	363	0.002

ENSP00000415615	CSNK2B	362	0.037
ENSP00000417404	HFE	794	0.015
ENSP00000419599	DPH3	363	0
ENSP00000420168	GSTA2	20	0
